# Beyond dissemination criteria: lesion-based longitudinal MRI of atypical demyelinating lesions at the boundary of multiple sclerosis

**DOI:** 10.1007/s00415-026-13951-6

**Published:** 2026-07-01

**Authors:** Yuriko Aratake, Kenji Yoshinaga, Shinji Ashida, Chihiro Fujii, Mio Hamatani, Kimitoshi Kimura, Hirofumi Ochi, Ryosuke Takahashi, Takayuki Kondo

**Affiliations:** 1 Department of Neurology, Kyoto University Graduate School of Medicine, Kyoto, Japan; 2 Department of Neurology, Kansai Medical University Medical Center, Osaka, Japan; 3 Department of Integrated Neuroanatomy and Neuroimaging, Kyoto University Graduate School of Medicine, Kyoto, Japan; 4 Department of Neurology, Kyoto Prefectural University of Medicine, Kyoto, Japan; 5 Department of Neurology and Geriatric Medicine, Ehime University Graduate School of Medicine, Ehime, Japan; 6 Kyoto University Research Administration Center, Kyoto, Japan

**Keywords:** Multiple sclerosis, Atypical inflammatory demyelinating lesions, Magnetic resonance imaging, Lesion phenotype, Longituditnal study, Therapeutical responce

## Abstract

**Background:**

Revisions of the McDonald diagnostic criteria have improved the sensitivity of multiple sclerosis (MS) diagnosis by incorporating MRI-based dissemination in space and time. However, some inflammatory demyelinating lesions fulfill dissemination criteria while showing radiological features atypical for MS, even in the absence of anti-AQP4 and anti-MOG antibodies. The long-term radiological course of these atypical idiopathic inflammatory demyelinating lesions (AIIDLs) remains unclear.

**Methods:**

We retrospectively analyzed 39 Japanese patients with AIIDLs, classified into multiple spotty lesions (MSLs), disseminated encephalomyelitis-like lesions (DEMLs), leukoencephalopathy-like lesions (LELs), and tumefactive lesions (TLs), and compared them with 97 patients with radiologically typical MS. Longitudinal MRI analyses evaluated lesion morphology, distribution, volume, and central vein sign (CVS) positivity, together with clinical outcomes and treatment responses.

**Results:**

Over a mean follow-up of 11 years, 38 of 39 AIIDL cases (97.4%) retained radiologically atypical features despite clinical relapses and fulfillment of dissemination criteria. MSLs and TLs lacked canonical MS features, such as periventricular lesions and T1 hypointense black holes. Only one case with LELs evolved into typical MS. CVS positivity exceeding 40% was observed in two MSLs cases but lesions remained atypical in distribution. Lesion volumes differed significantly among subgroups, with smaller volumes in MSLs and larger volumes in LELs compared with typical MS. Corticosteroids, immunosuppressants, and B cell depletion therapies appeared to be associated with clinical stabilization in several AIIDL subtypes, whereas conventional MS disease-modifying therapies were less consistently effective.

**Conclusions:**

In this cohort, most AIIDLs retained radiologically atypical brain MRI features over long-term follow-up despite recurrent inflammatory demyelinating events. These findings suggest that dissemination-based criteria alone may not fully capture the radiological heterogeneity of AQP4-IgG- and MOG-IgG-negative demyelinating disorders. Longitudinal lesion-based MRI phenotyping may provide complementary information for diagnostic reassessment although external validation and integration with optic nerve, spinal cord, and biological markers are required.

**Supplementary Information:**

The online version contains supplementary material available at 10.1007/s00415-026-13951-6.

## Introduction

Multiple sclerosis (MS) is a chronic inflammatory demyelinating disease of the central nervous system characterized by clinical, radiological, and biological heterogeneity [1]. Successive revisions of the McDonald diagnostic criteria have increased diagnostic sensitivity by incorporating MRI-based concepts of lesion dissemination, enabling earlier diagnosis and treatment initiation [1–4]. However, some patients with inflammatory demyelinating disorders present with clinical or radiological features that are atypical for conventional MS, and their evaluation remains challenging [5–7].

MRI plays a central role in refining MS diagnosis, and typical MS lesions show characteristic distributions, including periventricular, juxtacortical, infratentorial, and spinal cord involvement, as well as characteristic morphological features [8–12].

However, the boundary between radiologically typical and atypical demyelination remains insufficiently defined. This issue is particularly relevant in borderline cases, where patients diagnosed with MS may show heterogeneous radiological and immunological features [13–21].

In our previous studies [22, 23], we identified a subset of patients diagnosed and managed within the MS framework who exhibited radiological features atypical for conventional MS and who were treated more frequently with corticosteroids or other immunosuppressive therapies rather than conventional MS disease-modifying drugs (MS-DMDs). Their cerebrospinal fluid profiles also differed from those of typical MS [23]. These findings suggested that dissemination-based diagnostic frameworks may encompass patients with distinct radiological and clinical characteristics and that lesion phenotype might inform therapeutic decision-making.

Several studies have examined long-term outcomes of atypical demyelinating syndromes and clinically isolated demyelinating events [16, 18, 20, 24–27]. However, progression in these studies was typically defined by recurrent attacks and fulfillment of dissemination criteria; whether atypical lesions themselves evolve into radiologically typical MS lesions during long-term-follow-up has not been systematically examined, and little is known about how radiological phenotype may relate to therapeutic response.

To address these gaps, we performed a longitudinal MRI study of atypical idiopathic inflammatory demyelinating lesions (AIIDLs), defined as AQP4-IgG- and MOG-IgG-negative inflammatory demyelinating lesions with radiological features atypical for conventional MS. Importantly, AIIDLs are conceptualized as inflammatory demyelinating lesions located at the periphery of current MS diagnostic frameworks.

We compared these patients with radiologically typical MS and investigated whether atypical lesion phenotypes converge toward typical MS imaging patterns or remain stable over time. Because treatment decisions in clinical practice often hinge on MRI findings, we also analyzed treatment responses across lesion phenotypes. Our aim was to determine whether longitudinal lesion-based MRI phenotyping could provide complementary information for diagnostic reassessment and therapeutic decision-making beyond dissemination criteria alone.

## Methods

### Study design and participants

This retrospective cohort study included patients referred to a specialized MS clinic between October 2016 and December 2022. Eligible participants were patients with disease onset between 16 and 50 years of age who were diagnosed and managed within the MS diagnostic framework and demonstrated either radiologically typical MS or AIIDLs on brain MRI.

Patients with alternative diagnoses were excluded, including AQP4-IgG-positive NMOSD, MOG antibody-associated disease, systemic vasculitis, hereditary leukodystrophies, central nervous system infections, and other non-demyelinating neurological disorders. Patients without discrete white matter lesions or with MRI scans of insufficient quality due to significant motion artifacts were also excluded. Patients with longitudinally extensive spinal cord lesions, defined as lesions extending over three or more vertebral segments, were excluded from the study. Demographic and clinical data collected included age at disease onset, sex, disease duration, number of clinical relapses, and expanded disability status scale (EDSS) score at the last follow-up [28]. Laboratory assessments comprised cerebrospinal fluid oligoclonal bands (OCBs), serum autoimmune antibody panels, and other relevant biomarkers obtained as part of routine clinical evaluation. Detailed treatment history was recorded, with particular attention to the use of corticosteroids, conventional immunosuppressants, and MS-DMDs.

Serum AQP4-IgG and MOG-IgG were assessed using cell-based assays, and patients positive for either antibody were excluded. Antibody testing was repeated when clinically indicated in patients with features suggestive of NMOSD or MOGAD.

### Classification of demyelinating lesions

Demyelinating lesions were classified on the basis of baseline brain MRI findings. Patients were first categorized as having radiologically typical MS or AIIDLs according to overall lesion distribution, morphology, and radiological features. The AIIDL group was subsequently sub-classified into four lesion-based phenotypes reflecting distinct radiological patterns: multiple spotty lesions (MSLs), disseminated encephalomyelitis-like lesions (DEMLs), leukoencephalopathy-like lesions (LELs), and tumefactive lesions (TLs). This classification was based on lesion size, number, spatial distribution, and morphological characteristics observed on MRI. Detailed criteria for each subtype are provided in Table 1.

**Table 1:** Classification of atypical idiopathic inflammatory demyelinating lesions

Subtype	Definition
Multiple spotty lesions (MSLs)	Multiple round-shaped lesions < 5 mm in diameter located around the lateral ventricles in the white matter, without direct contact with the lateral ventricles or the callosal junction
Disseminated encephalomyelitis-like lesions (DEMLs)	Multiple asymmetrically disseminated lesions of unequal size (5–20 mm and >20 mm in diameter), located in the white matter with irregular margins including atypical morphology, and/or atypical location (e.g., wedge-shaped appearance), and/or atypical Gd- enhancement. This group also included cases in which lesions shrunk or resolved with the course of time, and cases with a focal lesion in the WM at onset and subsequent disseminated lesions
Leukoencephalopathy-like lesions (LELs)	Bilateral and symmetrical WM T2 hyper-intensities at first clinical onset, mimicking an inherited leukoencephalopathy or secondary progressive MS
Tumefactive lesions (TLs)	Tumor- or mass-like white matter-dominant solitary lesion exceeding 20 mm in size. This group also included cases in which the lesion shrunk or resolved with treatment

### MRI post-processing and central vein sign assessment

FLAIR* images were generated by co-registration followed by voxel-wise multiplication of high-resolution three-dimensional T2*-weighted echo-planar imaging and three-dimensional T2-FLAIR images. CVS assessment was performed in cases with MSLs in accordance with recommendations from the North American Imaging in Multiple Sclerosis Cooperative (NAIMS) [29–31]. Susceptibility-weighted imaging (SWI) datasets were reviewed in three orthogonal planes to evaluate the presence of the CVS using recently published consensus guidelines [32–34]. A central vein was considered present when it met all of the following criteria: a small apparent diameter (< 2 mm); appearance as a thin hypo-intense line or small hypo-intense dot; visibility in at least two perpendicular planes, with a linear configuration in at least one plane; partial or complete traversal of the lesion; and approximate localization at the center of the white matter lesion. For quantitative interpretation, lesions were classified as CVS-positive or CVS-negative, and the proportion of CVS-positive lesions was calculated for each case. A threshold of ≥ 40% CVS-positive lesions was considered supportive of MS-related pathology, consistent with previous reports [35].

### Spinal cord lesion assessment

Spinal cord lesions were also assessed on MRI [10]. MS-typical spinal cord lesions were characterized as focal, short-segment T2-hyperintense lesions located predominantly in the peripheral white matter, particularly within the dorsal or lateral columns, with an asymmetric distribution and limited cross-sectional involvement. Features considered atypical for MS included centrally located lesions, predominant gray matter involvement, longitudinally extensive lesions, and lesions occupying most of the spinal cord cross-sectional area.

### Data review and diagnostic evaluation

All clinical and imaging data were independently reviewed by four board-certified physicians, including one neuroradiologist and three neuro-immunology specialists, who were blinded to treatment status. Final classifications were determined by consensus, and any disagreements were resolved through discussion. Follow-up brain MRI scans were assessed for radiologic evolution, defined as the emergence of new lesions exhibiting radiologic features typical of MS. Therapeutic response was evaluated using a composite of clinical relapse occurrence, changes in expanded disability status scale (EDSS) score [28], and radiologic stability over time.

### Standard protocol approvals, registrations, and patient consents

This study was approved by the ethics committees of Kansai Medical University Medical Center. Written informed consent was obtained from all participants, and the study was conducted in accordance with the principles of the World Medical Association Declaration of Helsinki.

## Results

A total of 97 patients with radiologically typical MS and 39 patients with AIIDLs were included in the analysis. Based on baseline brain MRI findings, AIIDLs were sub-classified into multiple spotty lesions (MSLs; n = 18), disseminated encephalomyelitis-like lesions (DEMLs; n = 14), leukoencephalopathy-like lesions (LELs; n = 4), and tumefactive lesions (TLs; n = 3). Detailed definitions of each lesion phenotype are provided in Table 1. During a mean observation period of 11 years, new brain lesions were detected in 29 patients. Of these, 28 patients (16 with MSLs, 9 with DEMLs, 1 with LELs, and 2 with TLs) developed additional lesions that retained atypical radiological characteristics. In contrast, radiological conversion to MS-typical lesions was observed in only one patient with LELs during follow-up.

In addition, ten patients (two with MSLs, five with DEMLs, two with LELs, and one with TLs) showed no new brain lesions during follow-up, and their lesions remained radiologically atypical throughout the observation period. A marked reduction of pre-existing lesions was observed in five patients (two with MSLs, two with DEMLs, and one with TLs). Clinical characteristics and treatment strategies at the last follow-up are summarized in Table 2.

**Table 2:** Clinical and demographic characteristics of patients with atypical idiopathic inflammatory demyelinating lesions

No	Age group	sex	OCB	MBP	IgG index	Spinal cord lesions	ON	Gd	EDSS	treatment	lesion volume (mm^3^)
Clinical and demographic characteristics of 18 patients with MSLs											
1	30	F	+	N/A	N/A	C	+	-	1.5	NTZ	147.672803
2	50	F	−	+	N/A	C, T	−	−	0	none	114.631653
3	30	F	+	−	0.5 <	−	−	−	0	none	N/A
4	40	F	+	−	0.5 >	−	+	N/A	2	PSL + AZA	53.0481348
5	30	M	+	+	N/A	−	−	−	0	OFM	367.987061
6	30	F	-	+	N/A	C	−	−	0	PSL	29.2420395
7	30	F	+	−	0.5≦	C	−	+	6	OFM + PSL + TAC	128.534105
8	40	F	+	−	0.5≦	−	−	−	1.5	OFM + PSL + AZA	67.9254
9	50	F	+	N/A	N/A	−	−	−	1	OFM + MTX	67.3572237
10	40	F	−	−	0.5≦	−	+	−	1	OFM	93.4302835
11	30	F	−	−	0.5≦	T	+	N/A	3	OFM	115.871148
12	50	M	N/A	N/A	N/A	C	−	−	0	none	N/A
13	60	F	N/A	N/A	N/A	T	−	−	0	none	N/A
14	50	F	-	N/A	0.5≦	-	−	−	0	none	N/A
15	40	F	+	−	0.5≦	-	−	−	0	none	41.3060199
16	20	M	+	−	0.5≦	C	−	N/A	1	infliximab	N/A
17	40	F	−	N/A	0.5≦	−	+	−	1	DMF	N/A
18	40	M	−	N/A	N/A	−	+	−	3.5	OFM	82.206726
Clinical and demographic characteristics of 14 patients with DEMLs											
19	30	F	−	+	0.5≦	T	−	−	1	PSL + AZA	N/A
20	40	F	−	−	0.5≦	C	+	−	1	PSL + AZA	N/A
21	50	F	−	−	0.5≦	-	−	−	1	DMF	490.378874
22	40	F	+	N/A	0.5≦	C	N/A	−	8	OFM	N/A
23	40	M	-	+	0.5≦	-	+	+	4.5	OFM + PSL	N/A
24	20	M	−	−	N/A	-	−	−	2	OFM + PSL + CYA	344.374187
25	40	F	−	−	0.5 >	T	+	-	3	OFM	177.859314
26	40	F	−	−	N/A	C	-	-	1	OFM + PSL	255.310541
27	50	F	−	−	0.5≦	C	N/A	−	5.5	OFM	982.099793
28	60	F	−	N/A	0.5≦	T	−	+	5.0	PSL + AZA	N/A
29	30	M	−	N/A	0.5≦	−	−	−	1	PSL + AZA	N/A
30	20	F	−	N/A	0.5 >	C	−	+	0	OFM	N/A
31	40	F	+	N/A	N/A	−	−	−	1.5	NTZ	N/A
32	50	F	−	N/A	0.5≦	C	−	−	7.5	TAC	N/A
Clinical and demographic characteristics of four patients with LELs											
33	40	M	−	N/A	0.5≦	−	−	N/A	1	PSL + TAC	3750.28062
34	30	F	+	N/A	0.5≦	−	−	N/A	1	NTZ	1541.1377
35	40	M	+	N/A	0.5≦	C	+	N/A	7	DMF	7584.50644
36	40	F	+	-	N/A	C	+	-	8	OFM	706.398503
Clinical and demographic characteristics of three patients with TL											
37	60	F	−	N/A	0.5 >	−	+	−	2.5	PSL	N/A
38	40	M	−	−	0.5≦	C + T	−	+	6.5	OFM + PSL	727.379339
39	20	F	−	−	0.5 >	−	−	+	1	OFM + PSL	622.212901

*OCB* oligoclonal bands, *MBP* myelin basic protein, *ON* optic neuritis, *Gd* gadolinium enhancement, *EDSS* Expanded Disability Status Scale, *C* cervical spinal cord lesion, *T* thoracic spinal cord lesion, *PSL* prednisolone, *AZA* azathioprine, *OFM* ofatumumab, *NTZ* natalizumab, *DMF* dimethyl fumarate, *TAC* tacrolimus, *MTX* methotrexate, *CYA* cyclosporine, *N/A* not available, Symbols “ + ” and “ − ” indicate presence and absence, respectively

### Typical radiological presentations of MS

**Fig. 1:**
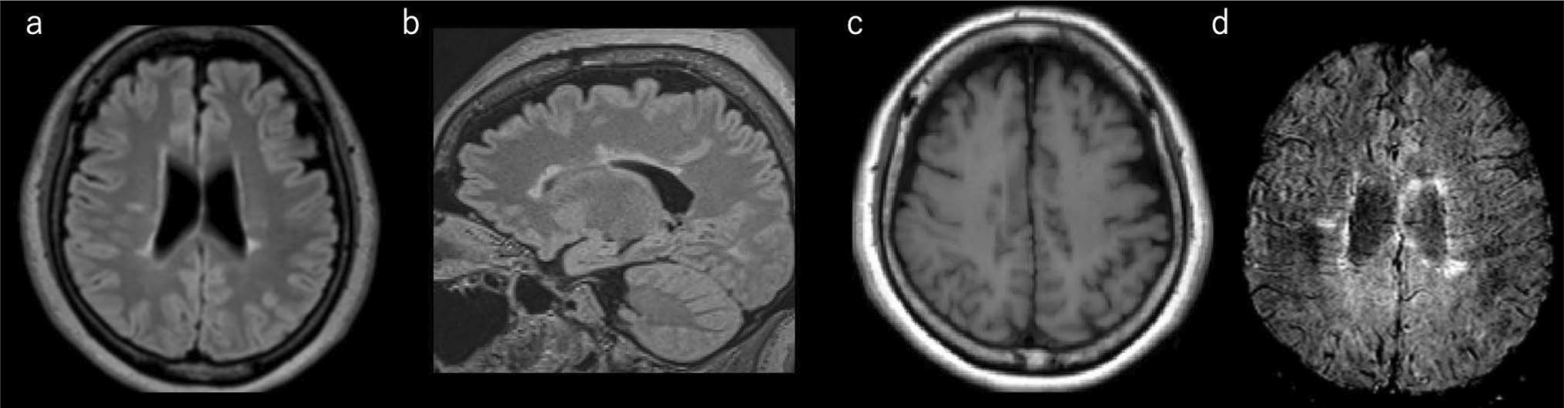
Representative magnetic resonance imaging (MRI) features of radiologically typical multiple sclerosis (MS). Axial brain MR images from a 48-year-old woman (case 3) with relapsing–remitting MS, Fluid-attenuated inversion recovery (FLAIR) images (**a, b**) show ovoid periventricular lesions (**a**) and a Dawson’s finger projection (**b**). A T1-weighted image (**c**) demonstrates a hypo-intense “black hole” lesion. A susceptibility-weighted FLAIR image (**d**) reveals a central vein sign within a white matter lesion. These radiological features are consistent with a typical MS phenotype

A total of 97 patients exhibited radiologically typical features of MS. The median age at disease onset was 28.5 years (range 18–47 years), and the median age at the end of follow-up was 47 years (range 26–71 years). The median EDSS score at the last follow-up was 3.0 (range 0–9), and the median disease duration was 15.7 years (range 2–43 years). The annualized relapse rate (ARR) was 0.37. All patients demonstrated periventricular lesions, defined as lesions distributed along the bodies of the lateral ventricles on FLAIR images (Fig. 1). Gadolinium enhancement was observed in 16% of cases during follow-up. Lesion volume analysis was performed in a subset of 25 patients, revealing a median lesion volume of 347.137 mm^3^ (range 65.205–11,170.525 mm^3^).

### Multiple spotty lesions group

Eighteen patients were classified as having MSLs. The median age at disease onset was 34.5 years (range 21–48 years), and the median age at the end of follow-up was 44 years (range 29–60 years). The median EDSS score at the last follow-up was 0.5 (range 0–6), and the median disease duration was 9 years (range 2–25 years). In all cases, lesions were exclusively supratentorial, with no infratentorial involvement. Clinical symptoms were similar to those observed in typical MS. The ARR was 0.082. OCBs in cerebrospinal fluid were detected in nine cases (1, 3–5, 7–9, 15, and 16), while an elevated IgG index was observed in nine cases (3, 7, 8, 10, 11, and 14–17).

**Fig. 2:**
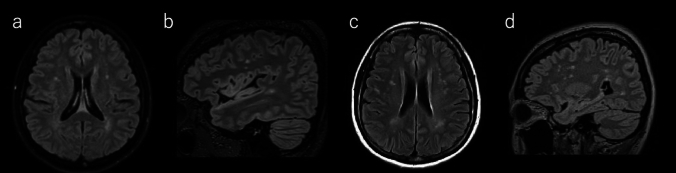
Representative case of multiple spotty lesions. Brain magnetic resonance images from case 8 at initial presentation (**a, b**) and at follow-up 6 years after onset (**c, d**). Axial Fluid-attenuated inversion recovery (FLAIR) images (**a, c**) and sagittal FLAIR images (**b, d**) demonstrate small, discrete white matter lesions located in the periventricular region but not abutting the lateral ventricles. Lesions remained non-confluent and radiologically atypical over time

On MRI, lesions were small and discrete, with a median lesion volume of 87.819 mm^3^ (range 29.242–367.987 mm^3^). Lesions showed no confluence and did not exhibit T1 hypo-intense black holes, in contrast to radiologically typical MS (Fig. 2). The mean ratio of periventricular lesions to all white matter lesions was zero.

CVS analysis was performed in MSL cases. Using the ≥ 40% CVS-positive lesion threshold (“40% rule”) [35], two patients (cases 8 and 15) met this criterion. However, in both cases, lesions were located away from the lateral ventricles. Case 8 did not respond to MS-DMDs other than B cell depletion therapy. Regarding treatment response, two of five patients (40%) treated with corticosteroids or immunosuppressants experienced relapse, whereas seven of nine patients initially treated with MS-DMDs other than B cell depletion relapsed (odds ratio 5.25, *p* = 0.266).

### Disseminated encephalomyelitis-like lesions group

**Fig. 3:**
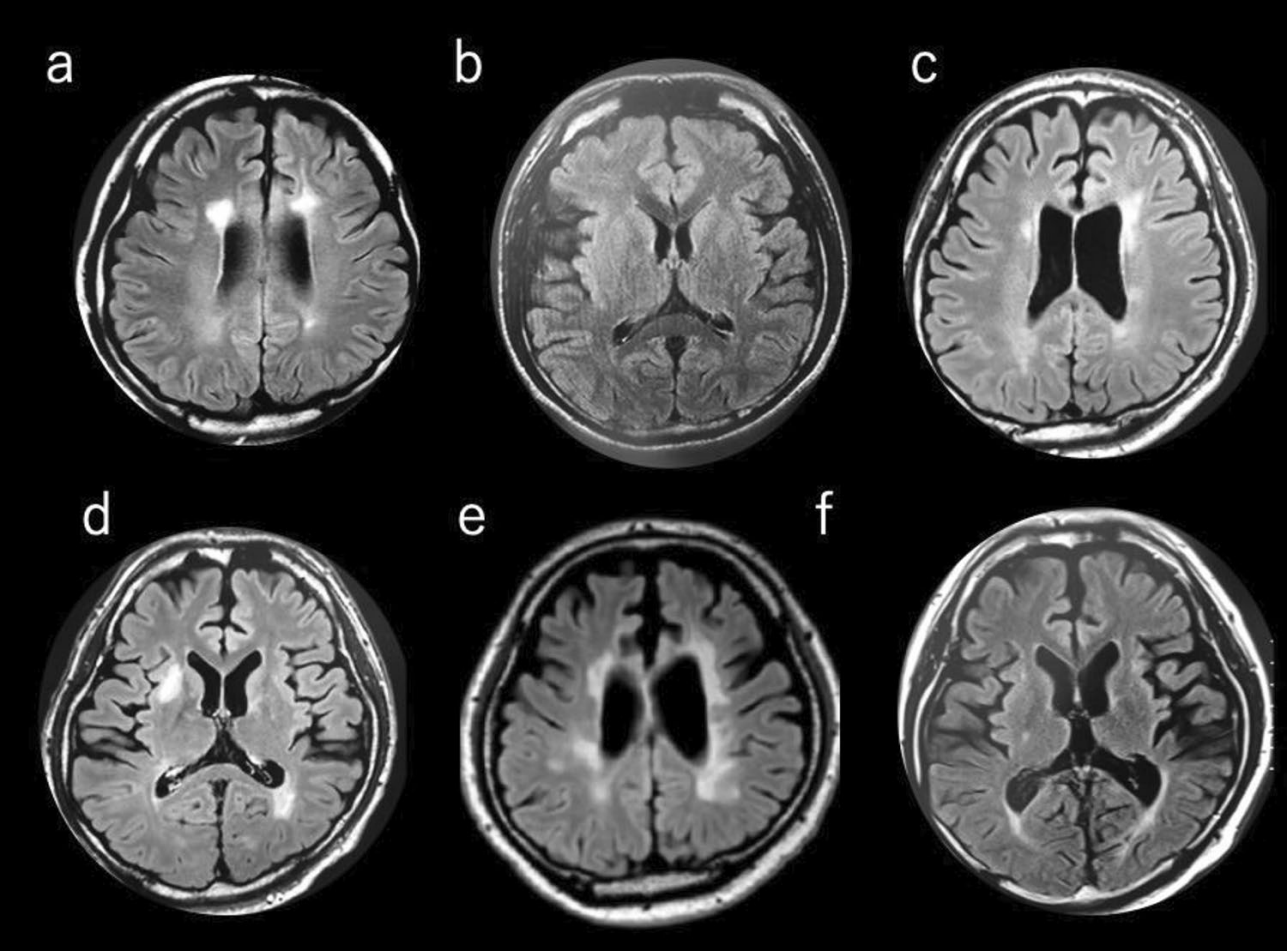
Representative case of disseminated encephalomyelitis-like lesions. Brain magnetic resonance (MR) images from case 23, exhibiting atypical features in lesion location, morphology, and gadolinium (Gd) enhancement pattern. Initial MRI at onset (**a,b**) shows multiple edematous lesions involving the periventricular white matter (**b**) with irregular, jagged Gd enhancement. Three years later (**c, d**), new disseminated lesions appeared in the bilateral centrum semiovale, right basal ganglia (**d**), and pons. At 10-year follow-up (**e, f**), brain atrophy became evident, and confluent lesions were observed in the bilateral centrum semiovale, periventricular white matter (**e**), and middle cerebellar peduncles. Although some lesions were located in the periventricular white matter, this case was classified as disseminated encephalomyelitis-like lesions because the lesions showed atypical morphology for conventional MS, including relatively large and edematous lesions at initial presentation (**a, b**), involvement of the right basal ganglia during follow-up (**d**), and regression or disappearance of some pre-existing lesions over time (**e**)

14 patients were classified as having DEMLs. The median age at disease onset was 36 years (range 20–50 years), and the median age at the end of follow-up was 45.5 years (range 24–63 years). The median EDSS score at the last follow-up was 1.5 (range 0–7.5), with a median disease duration of 12.5 years (range 1–25 years). The ARR was 0.101. Fever or headache at onset, atypical for MS attacks, was observed in cases 11 and 13, while none of the patients exhibited impaired consciousness. The median lesion volume was 344.374 mm^3^ (range 177.859–982.010 mm^3^). Based on lesion distribution and morphology, DEMLs were further categorized into three subgroups: MS-typical location with atypical morphology (*n* = 7; cases 20, 21, 22, 25, 26, 27, and 32), MS-atypical location and morphology (*n* = 4; cases 19, 24, 28, and 31), and MS-atypical location, morphology, and enhancement (*n* = 3; cases 23, 29, and 30) (Fig. 3). Cerebrospinal fluid OCBs were present in two cases (22 and 31), and the IgG index was elevated in nine cases (19–23, 27–29, and 32).

Five patients (cases 19, 25, 28, 29, and 32) developed no new lesions during follow-up. The remaining nine patients developed new lesions; however, these lesions retained atypical radiological characteristics. Complete disappearance of pre-existing lesions was observed in two cases (19 and 31). Notably, none of the patients developed radiologically typical MS lesions during follow-up.

Regarding treatment, two patients remained clinically stable without therapy, while five were treated with corticosteroids or immunosuppressants. Two of these relapsed but subsequently stabilized after initiation of B cell depletion therapy. Conversely, three of four patients initially treated with MS-DMDs other than B cell depletion relapsed but stabilized after switching to B cell depletion therapy. All patients tested negative for autoimmune antibodies associated with collagen diseases, as well as for anti-AQP4 antibodies and anti-MOG antibodies.

### Leukoencephalopathy-like lesions group

Four patients were classified as having LELs, including two women and two men. The median age at symptom onset was 25.5 years (range 16–44 years), and the median age at the last follow-up was 45.5 years (range 33–54 years). Cerebrospinal fluid OCBs were detected in three cases (cases 34–36), and an elevated IgG index was observed in three cases (cases 33–35). The median number of clinical attacks was five (range 2–12). The median EDSS score at the last follow-up was 4.75 (range 1.0–7.0), and the median disease duration was 13 years (range 10–31 years). The ARR was 0.68. All patients were negative for autoimmune antibodies associated with connective tissue diseases, as well as anti-AQP4 and anti-MOG antibodies.

LELs were characterized by large lesion volumes, with a median of 2645.709 mm^3^ (range 706.399–7584.506 mm^3^).

All patients presented with clinical symptoms resembling typical MS. At follow-up, natalizumab was administered in case 34, dimethyl fumarate in case 35, and ofatumumab in case 36.

**Fig. 4:**
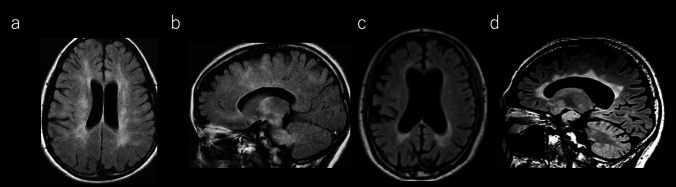
Representative case of leukoencephalopathy-like lesions. Brain magnetic resonance images from case 34 at two time points. Axial Fluid-attenuated inversion recovery images at onset (**a, b**) and 15 years after symptom onset (**c, d**) show the evolution of white matter lesions. Initially, the lesions were ill-defined and bilaterally distributed (**a, b**). At follow-up (**c, d**), the lesion boundaries became well-demarcated and vertically oriented along the lateral ventricles, consistent with Dawson’s fingers. This case demonstrates radiological conversion to a typical multiple sclerosis pattern over time

Case 34 showed radiological conversion to typical MS (Fig. 4) and responded favorably to natalizumab. The remaining three patients retained LEL-type features throughout follow-up. Case 35 demonstrated clinical improvement with dimethyl fumarate despite the persistence of LEL-type MRI features.

### Tumefactive lesions group

Three patients were classified as having TLs, including two women and one man. The median age at onset was 32 years (range 25–50 years), and the median age at the last follow-up was 43 years (range 28–62 years). The median number of clinical attacks was two (range 2–3), and the median disease duration was 12 years (range 4–13 years). The median EDSS score at the last follow-up was 2.5 (range 1–6.5). The ARR was 0.050. All patients were negative for autoimmune antibodies associated with connective tissue diseases, as well as anti-AQP4 and anti-MOG antibodies. All TL cases were negative for cerebrospinal fluid OCBs. Case 38 showed an elevated IgG index.

**Fig. 5:**
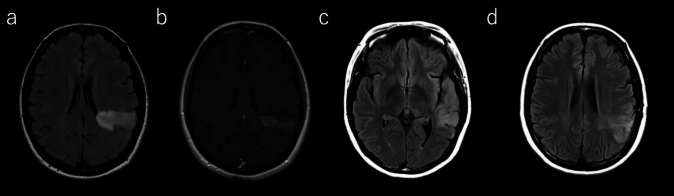
Representative case of a tumefactive lesion. Brain magnetic resonance images from case 39. Initial Fluid-attenuated inversion recovery (**a**) and T1-weighted gadolinium-enhanced image (**b**) show a large tumefactive lesion involving the left white matter and adjacent cortex. Six months later, the lesion regressed spontaneously. At 2-year follow-up (**c, d**), a new focal edematous lesion appeared in the left temporal lobe (**c**) without gadolinium enhancement. After treatment with intravenous corticosteroids, plasma exchange, and intravenous immunoglobulin, the lesion resolved

Clinical symptoms in cases 37 and 38 differed from typical MS attacks. The median lesion volume was 622.213 mm^3^ (range 499.737–727.379 mm^3^). Brain biopsy in cases 38 and 39 confirmed perivascular lymphocytic infiltration and demyelination. Although lesions regressed or resolved with treatment, two patients subsequently relapsed with atypical demyelinating lesions on MRI (Fig. 5c). At follow-up, one patient was treated with prednisolone alone, while two received combined ofatumumab and prednisolone therapy.

### Spinal cord assessment

Among cases with spinal cord lesions, MS-typical lesions were observed in cases 1, 16, 22, and 25, whereas all other assessable cases showed atypical spinal cord lesions. These included gray matter-dominant lesions in cases 2, 6, 7, 12, 27, and 32, centrally located lesions in cases 11, 26, 30, 35, and 36, and lesions occupying a relatively large portion of the spinal cord cross-sectional area in cases 20, 28, and 38. Spinal cord MRI was not available for cases 13 and 19.

### Volumetric comparison between subgroups

**Fig. 6:**
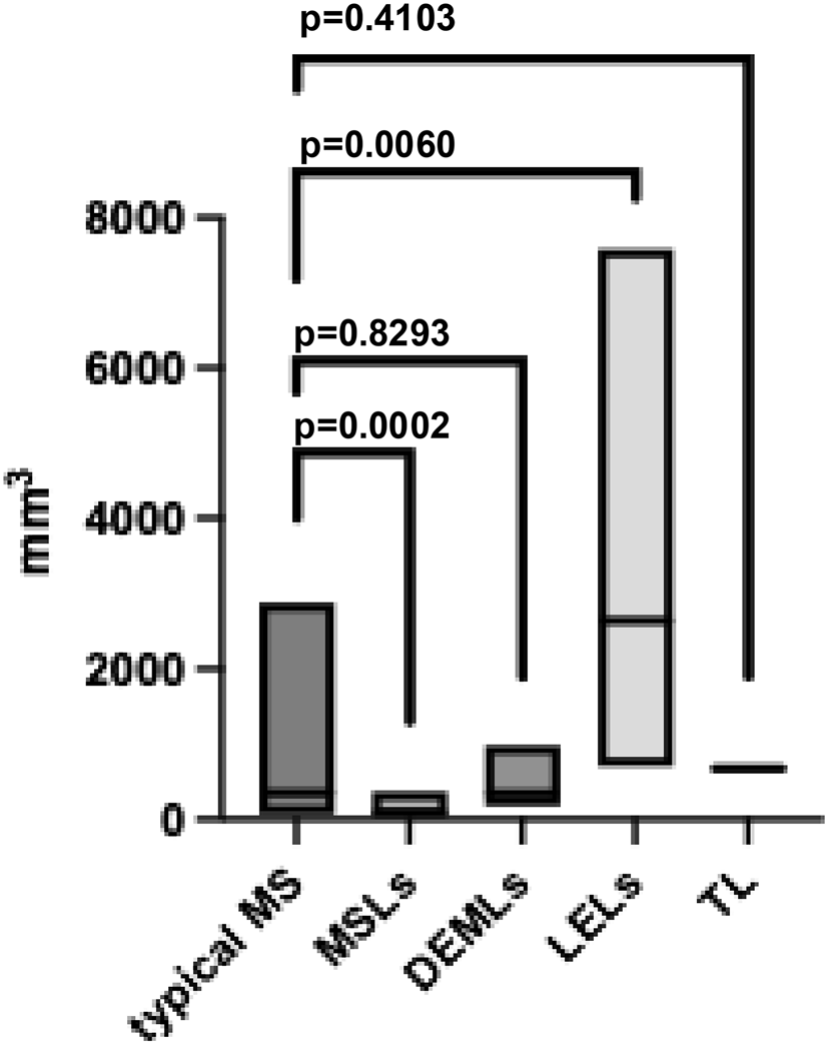
Comparison of Brain Lesion Volumes Among Radiologically Typical multiple sclerosis (MS), multiple spotty lesions (MSLs), disseminated encephalomyelitis-like lesions (DEMLs), leukoencephalopathy-like lesions (LELs), and tumefactive lesions (TLs). Boxplots displaying lesion volume distributions (mm^3^) across five groups: radiologically typical MS, MSLs, DEMLs, LELs, and TLs. Each box shows the median, interquartile range, and full range, with individual data points overlaid. Lesion volumes were calculated from 3D-FLAIR MRI images using manual segmentation in FSLeyes. MSLs demonstrated significantly smaller lesion volumes compared to typical MS (*p* < 0.0001), while LELs showed significantly larger volumes (*p* = 0.0060). No significant differences were found between typical MS and DEMLs or TLs. Statistical comparisons were conducted using the Mann–Whitney U test

Lesion volume analysis revealed distinct patterns across lesion-based subgroups (Fig. 6). MSLs demonstrated the smallest median lesion volume (87.819 mm^3^), which was significantly lower than that observed in radiologically typical MS (median 347.137 mm^3^; *p* = 0.0002). In contrast, LELs were characterized by markedly larger lesion volumes (median 2645.7 mm^3^; *p* = 0.0060) compared with typical MS.

No statistically significant differences in lesion volume were observed between typical MS and DEMLs (median 344.374 mm^3^; *p* = 0.8293) or TLs (median 622.213 mm^3^; *p* = 0.4103). These comparisons were limited by the small number of cases within these subgroups.

## Discussion

In this longitudinal cohort study of 39 Japanese patients with AIIDLs, we found that 97.4% of cases consistently exhibited radiologically atypical features throughout the disease course despite meeting both dissemination in space and dissemination in time criteria. These findings suggest that classifying and managing all demyelinating disorders fulfilling dissemination criteria in the same way as conventional MS may not be appropriate and reinforce the importance of continuous diagnostic reassessment in atypical presentations. Previous studies have described the long-term outcomes of atypical or clinically isolated demyelinating events [18, 21, 36]; however, when follow-up examinations revealed relapses and fulfillment of dissemination criteria alone, such cases were typically judged as MS. In contrast, the longitudinal radiological evolution of atypical inflammatory demyelinating lesions—specifically whether their MRI characteristics eventually resemble those typical of MS—has not been well established.

By systematically tracking lesion morphology, distribution, and volume over time, our study provides a lesion-centered longitudinal analysis that differs from prior approaches.

Among the lesion-based phenotypes, MSLs exhibited several features atypical for MS, including the absence of T1 hypo-intense black holes, lack of lesion confluence, low periventricular lesion burden, and significantly smaller lesion volumes. Although MSLs have not been emphasized in prior reports of AIIDLs, atypical MS characteristics are often overlooked in routine clinical practice, and patients with MSLs are frequently referred with a diagnosis or suspicion of MS. Notably, two MSL cases fulfilled the ≥ 40% CVS threshold. However, CVS positivity alone is insufficient to establish a diagnosis of MS [32–34], particularly when lesion distribution and morphology are atypical for conventional MS. Rather, CVS should be interpreted in combination with lesion topography, morphology, clinical presentation, CSF findings, antibody testing, and longitudinal radiological evolution. Our findings therefore do not challenge the diagnostic value of CVS but instead emphasize that CVS should not be used as a standalone marker in diagnostically ambiguous cases.

Moreover, the presence of autoantibodies associated with collagen vascular diseases in several MSL cases suggests that these lesions may share pathogenic mechanisms with systemic autoimmune or vasculitis processes rather than typical MS [37–39]. This interpretation is further supported by the favorable response to corticosteroids and immunosuppressants, and the limited efficacy of MS disease-modifying therapies other than B cell depletion therapy.

In our study, DEMLs also remained radiologically atypical over time. The relationship between acute disseminated encephalomyelitis and MS has long been controversial, particularly in adults [16, 21, 40–43]. While pediatric studies have reported progression from ADEM to MS based on fulfillment of dissemination criteria [42, 44], adult-onset cases are less well defined [45, 46]. Importantly, subsequent recognition of anti-MOG antibody-associated disease has revealed that a substantial proportion of pediatric cases previously classified as ADEM or pediatric MS actually represent a distinct disease entity [42, 47]. This historical reclassification underscores the potential risk of defining disease evolution solely by recurrent attacks and fulfillment of dissemination criteria, without consideration of underlying disease biology or lesion phenotype. Consistent with this perspective, previous studies assessing diagnostic progression have focused primarily on the appearance of new symptoms or lesions, without evaluating whether lesions acquired radiologically typical MS features [40, 46]. In our cohort, DEMLs exhibited clinical relapses but did not evolve into MS-typical imaging patterns. Complete resolution of pre-existing lesions, observed in two DEMLs cases, is rarely seen in typical MS and further supports distinct underlying pathophysiology. This interpretation is further supported by the observation that several DEMLs cases remained stable without disease-modifying therapy or under corticosteroids and immunosuppressants, whereas MS-DMDs other than B cell depletion were less effective, with disease stabilization achieved after B cell depletion therapy.

TLs similarly posed diagnostic challenges [16, 18, 21, 48–50]. Although a monophasic course has often been assumed, previous reports have described recurrent cases and progression to MS based on clinical and radiological recurrence [51, 52]. However, none of the tumefactive lesion cases in our cohort converted to radiologically typical MS, despite relapse. Lesion resolution during remission, heterogeneous contrast enhancement patterns, absence of OCBs, and favorable responses to corticosteroids further distinguish tumefactive lesions from typical MS and underscore the limitations of relying solely on dissemination criteria for diagnosis and management.

LELs exhibited a distinct pattern compared with other AIIDL subtypes [21]. Although their initial MRI appearance was clearly atypical for MS, showing large and diffuse lesions, their subsequent clinical course and treatment response partially overlapped with those of typical MS [21]. In one case, radiological conversion was observed. In addition, some patients with LELs showed cerebrospinal fluid OCBs, an important supportive biomarker in MS, and responded well to established MS-DMDs. These findings suggest that a subset of LELs may share immunopathological features with typical MS, although the small number of cases prevents definitive conclusions.

The advent of high-efficacy MS therapies has substantially improved outcomes in typical MS [53], yet their applicability to AIIDLs remains uncertain. Although treatment strategies in this cohort were not standardized, MSLs and DEMLs frequently showed insufficient responses to conventional MS-DMDs, whereas B cell depletion therapy was effective across multiple AIIDL subtypes. These observations, together with prior reports demonstrating distinct cytokine profiles and differential steroid responsiveness [22, 23], suggest that uniform application of MS-DMDs to atypical demyelinating lesions may be inappropriate. Instead, individualized therapeutic strategies based on lesion phenotype and presumed pathophysiology may be warranted. Recent studies have used advanced MRI and plasma metabolomic approaches to classify patients at the borderline between MS and NMOSD, suggesting that objective, multidimensional phenotyping may help identify biologically distinct demyelinating subgroups [54, 55]. In contrast, the present study was based mainly on longitudinal conventional brain MRI findings. Although lesion volumetry was included, advanced MRI metrics and molecular biomarkers were not available. Thus, our MRI-based subgroups should be interpreted as descriptive radiological categories, and future studies integrating standardized CNS-wide imaging and biomarker analyses are needed.

Several limitations should be acknowledged. First, the retrospective, single-center design and limited sample size, particularly in the LELs and TLs subgroups, limit the generalizability of our findings. In particular, because our institution serves as a referral center for diagnostically challenging inflammatory demyelinating disorders, patients with atypical radiological features may have been overrepresented, introducing potential referral bias. Accordingly, the prevalence and clinical implications of AIIDLs in this cohort should be interpreted with caution and may not be directly generalizable to broader MS populations.

Second, this study primarily focused on brain MRI findings. Although available spinal cord imaging data were reviewed, spinal cord lesions were not systematically assessed using a standardized protocol, and optic nerve involvement was not comprehensively evaluated. Thus, the proposed brain lesion-based classification may not fully reflect CNS-wide disease phenotypes. Furthermore, paramagnetic rim lesions were not systematically assessed because susceptibility imaging protocols were not optimized or standardized for PRL evaluation across the long observation period.

Third, although CVS assessment and volumetric analyses were performed rigorously, they relied on visual and semi-quantitative methods, which may introduce observer variability. The MRI-based subgroups proposed in this study lack external validation and should therefore be regarded as descriptive radiological categories rather than established diagnostic entities.

Finally, treatment observations in this cohort should be interpreted as hypothesis-generating, as treatment was not standardized and was influenced by disease severity, physician preference, and diagnostic uncertainty; therefore, no causal inference regarding therapeutic efficacy can be made. Future studies incorporating standardized brain, optic nerve, and spinal cord imaging protocols, independent neuroradiological review, and inter-rater agreement analyses are needed to establish a more comprehensive phenotyping framework and confirm the reproducibility of this classification.

In this cohort, most AIIDLs retained radiologically atypical brain MRI features over long-term follow-up. These findings do not establish AIIDLs as a distinct disease entity and do not exclude the possibility that some cases represent atypical forms of MS. Rather, they suggest that recurrent inflammatory demyelinating disorders evaluated within the MS diagnostic framework may show persistent radiological heterogeneity that is not fully captured by dissemination criteria alone. Longitudinal lesion-based MRI assessment may therefore provide complementary information for diagnostic reassessment. Future prospective multicenter studies incorporating standardized brain, spinal cord, and optic nerve imaging, independent imaging validation, advanced MRI markers, and biological biomarkers are required to clarify the pathological significance of these radiological phenotypes.

## Disclosure

The authors report no relevant disclosures.

## Supplementary Information

Below is the link to the electronic supplementary material.

Supplementary file1 (DOCX 25 KB)

## Data Availability

The datasets generated and/or analyzed during the current study are available from the corresponding author on reasonable request, in accordance with institutional and ethical regulations.

## References

[1] Schumacher GA, Beebe G, Kibler RF, Kurland LT, Kurtzke JF, McDowell F et al (1965) Problems of experimental trials of therapy in multiple sclerosis: report by the panel on the evaluation of experimental trials of therapy in multiple sclerosis. Ann N Y Acad Sci 122:552–568. 10.1111/j.1749-6632.1965.tb20235.x 14313512 10.1111/j.1749-6632.1965.tb20235.x

[2] Montalban X, Lebrun-Frénay C, Oh J, Arrambide G, Moccia M, Pia Amato M et al (2024) Diagnosis of multiple sclerosis: 2024 revisions of the McDonald criteria. Lancet Neurol 24(10):850–865. 10.1016/S1474-4422(25)00270-4 10.1016/S1474-4422(25)00270-4 40975101

[3] Thompson AJ, Banwell BL, Barkhof F, Carroll WM, Coetzee T, Comi G et al (2018) Diagnosis of multiple sclerosis: 2017 revisions of the McDonald criteria. Lancet Neurol 17(2):162–173. 10.1016/S1474-4422(17)30470-2 29275977 10.1016/S1474-4422(17)30470-2

[4] Poser CM, Brinar VV (2004) Diagnostic criteria for multiple sclerosis: an historical review. Clin Neurol Neurosurg 106(3):147–158. 10.1016/j.clineuro.2004.02.004 15177763 10.1016/j.clineuro.2004.02.004

[5] Solomon AJ, Corboy JR (2017) The tension between early diagnosis and misdiagnosis of multiple sclerosis. Nat Rev Neurol 13(9):567–572. 10.1038/nrneurol.2017.106 28799551 10.1038/nrneurol.2017.106

[6] Solomon AJ, Klein EP, Bourdette D (2012) “Undiagnosing” multiple sclerosis: the challenge of misdiagnosis in MS. Neurology 78(24):1986–1991. 10.1212/WNL.0b013e318259e1b2 22581930 10.1212/WNL.0b013e318259e1b2 PMC3369504

[7] Gaitán MI, Correale J (2019) Multiple sclerosis misdiagnosis: a persistent problem to solve. Front Neurol 10:466. 10.3389/fneur.2019.00466 31133970 10.3389/fneur.2019.00466 PMC6514150

[8] Barkhof F, Filippi M, Miller DH, Scheltens P, Campi A, Polman CH et al (1997) Comparison of MRI criteria at first presentation to predict conversion to clinically definite multiple sclerosis. Brain 120(Pt 11):2059–2069. 10.1093/brain/120.11.2059 9397021 10.1093/brain/120.11.2059

[9] Charil A, Yousry TA, Rovaris M, Barkhof F, De Stefano N, Fazekas F et al (2006) MRI and the diagnosis of multiple sclerosis: expanding the concept of “no better explanation.” Lancet Neurol 5(10):841–852. 10.1016/S1474-4422(06)70572-5 16987731 10.1016/S1474-4422(06)70572-5

[10] Filippi M, Preziosa P, Banwell BL, Barkhof F, Ciccarelli O, De Stefano N et al (2019) Assessment of lesions on magnetic resonance imaging in multiple sclerosis: practical guidelines. Brain 142(7):1858–1875. 10.1093/brain/awz144 31209474 10.1093/brain/awz144 PMC6598631

[11] Filippi M, Preziosa P, Arnold DL, Barkhof F, Harrison DM, Maggi P et al (2023) Present and future of the diagnostic work-up of multiple sclerosis: the imaging perspective. J Neurol 270(3):1286–1299. 10.1007/s00415-022-11488-y 36427168 10.1007/s00415-022-11488-y PMC9971159

[12] Geraldes R, Ciccarelli O, Barkhof F, De Stefano N, Enzinger C, Filippi M et al (2018) The current role of MRI in differentiating multiple sclerosis from its imaging mimics. Nat Rev Neurol 14(4):213. 10.1038/nrneurol.2018.39 29582852 10.1038/nrneurol.2018.39

[13] Juryńczyk M, Weinshenker B, Akman-Demir G, Asgari N, Barnes D, Boggild M et al (2016) Status of diagnostic approaches to AQP4-IgG seronegative NMO and NMO/MS overlap syndromes. J Neurol 263(1):140–149. 10.1007/s00415-015-7952-8 26530512 10.1007/s00415-015-7952-8 PMC4816597

[14] Alper G, Heyman R, Wang L (2009) Multiple sclerosis and acute disseminated encephalomyelitis diagnosed in children after long-term follow-up: comparison of presenting features. Dev Med Child Neurol 51(6):480–486. 10.1111/j.1469-8749.2008.03136.x 19018840 10.1111/j.1469-8749.2008.03136.x PMC2704249

[15] Cortese R, Battaglini M, Prados F, Bianchi A, Haider L, Jacob A et al (2022) Clinical and MRI measures to identify non-acute MOG-antibody disease in adults. Brain. 10.1093/brain/awac480 36515653 10.1093/brain/awac480

[16] Ayrignac X, Carra-Dallière C, Labauge P (2018) Atypical inflammatory demyelinating lesions and atypical multiple sclerosis. Rev Neurol (Paris) 174(6):408–418. 10.1016/j.neurol.2018.03.007 29673573 10.1016/j.neurol.2018.03.007

[17] Lucchinetti C, Brück W, Parisi J, Scheithauer B, Rodriguez M, Lassmann H (2000) Heterogeneity of multiple sclerosis lesions: implications for the pathogenesis of demyelination. Ann Neurol 47(6):707–717. 10.1002/1531-8249(200006)47:6<707::aid-ana3>3.0.co;2-q 10852536 10.1002/1531-8249(200006)47:6<707::aid-ana3>3.0.co;2-q

[18] Wallner-Blazek M, Rovira A, Fillipp M, Rocca MA, Miller DH, Schmierer K et al (2013) Atypical idiopathic inflammatory demyelinating lesions: prognostic implications and relation to multiple sclerosis. J Neurol 260(8):2016–2022. 10.1007/s00415-013-6918-y 23620065 10.1007/s00415-013-6918-y

[19] Koelblinger C, Fruehwald-Pallamar J, Kubin K, Wallner-Blazek M, van den Hauwe L, Macedo L et al (2013) Atypical idiopathic inflammatory demyelinating lesions (IIDL): conventional and diffusion-weighted MR imaging (DWI) findings in 42 cases. Eur J Radiol 82(11):1996–2004. 10.1016/j.ejrad.2013.07.026 23993757 10.1016/j.ejrad.2013.07.026

[20] Seewann A, Enzinger C, Filippi M, Barkhof F, Rovira A, Gass A et al (2008) MRI characteristics of atypical idiopathic inflammatory demyelinating lesions of the brain : A review of reported findings. J Neurol 255(1):1–10. 10.1007/s00415-007-0754-x 18004634 10.1007/s00415-007-0754-x

[21] Codjia P, Ayrignac X, Carra-Dalliere C, Cohen M, Charif M, Lippi A et al (2019) Multiple sclerosis with atypical MRI presentation: Results of a nationwide multicenter study in 57 consecutive cases. Mult Scler Relat Disord 28:109–116. 10.1016/j.msard.2018.12.022 30592992 10.1016/j.msard.2018.12.022

[22] Ashida S, Ochi H, Hamatani M, Fujii C, Nishigori R, Kawamura K et al (2021) Radiological and Laboratory Features of Multiple Sclerosis Patients With Immunosuppressive Therapy: A Multicenter Retrospective Study in Japan. Front Neurol 12:749406. 10.3389/fneur.2021.749406 34721276 10.3389/fneur.2021.749406 PMC8548818

[23] Ashida S, Kondo T, Fujii C, Hamatani M, Mizuno T, Ochi H (2022) Association of cerebrospinal inflammatory profile with radiological features in newly diagnosed treatment-naïve patients with multiple sclerosis. Front Neurol 13:1012857. 10.3389/fneur.2022.1012857 36203996 10.3389/fneur.2022.1012857 PMC9530286

[24] Al-Louzi O, Letchuman V, Manukyan S, Beck ES, Roy S, Ohayon J et al (2022) Central vein sign profile of newly developing lesions in multiple sclerosis: a 3-year longitudinal study. Neurol Neuroimmunol Neuroinflamm. 10.1212/NXI.0000000000001120 35027474 10.1212/NXI.0000000000001120 PMC8759076

[25] De Stefano N, Giorgio A, Tintoré M, Pia Amato M, Kappos L, Palace J et al (2018) Radiologically isolated syndrome or subclinical multiple sclerosis: MAGNIMS consensus recommendations. Mult Scler 24(2):214–221. 10.1177/1352458517717808 29451440 10.1177/1352458517717808

[26] Kepes JJ (1993) Large focal tumor-like demyelinating lesions of the brain: intermediate entity between multiple sclerosis and acute disseminated encephalomyelitis? A study of 31 patients. Ann Neurol 33(1):18–27. 10.1002/ana.410330105 8494332 10.1002/ana.410330105

[27] Liao MF, Huang CC, Lyu RK, Chen CM, Chang HS, Chu CC et al (2011) Acute disseminated encephalomyelitis that meets modified McDonald criteria for dissemination in space is associated with a high probability of conversion to multiple sclerosis in Taiwanese patients. Eur J Neurol 18(2):252–259. 10.1111/j.1468-1331.2010.03114.x 20561038 10.1111/j.1468-1331.2010.03114.x

[28] Kurtzke JF (1983) Rating neurologic impairment in multiple sclerosis: an expanded disability status scale (EDSS). Neurology 33(11):1444–1452. 10.1212/wnl.33.11.1444 6685237 10.1212/wnl.33.11.1444

[29] Sati P, Oh J, Constable RT, Evangelou N, Guttmann CR, Henry RG et al (2016) The central vein sign and its clinical evaluation for the diagnosis of multiple sclerosis: a consensus statement from the North American Imaging in Multiple Sclerosis Cooperative. Nat Rev Neurol 12(12):714–722. 10.1038/nrneurol.2016.166 27834394 10.1038/nrneurol.2016.166

[30] Solomon AJ, Watts R, Ontaneda D, Absinta M, Sati P, Reich DS (2018) Diagnostic performance of central vein sign for multiple sclerosis with a simplified three-lesion algorithm. Mult Scler 24(6):750–757. 10.1177/1352458517726383 28820013 10.1177/1352458517726383 PMC5794670

[31] Sinnecker T, Clarke MA, Meier D, Enzinger C, Calabrese M, De Stefano N et al (2019) Evaluation of the central vein sign as a diagnostic imaging biomarker in multiple sclerosis. JAMA Neurol 76(12):1446–1456. 10.1001/jamaneurol.2019.2478 31424490 10.1001/jamaneurol.2019.2478 PMC6704746

[32] Solomon AJ (2020) Progress towards a diagnostic biomarker for MS: Central vein sign. Mult Scler 26(4):394–396. 10.1177/1352458520907910 32249717 10.1177/1352458520907910

[33] Bhandari A, Xiang H, Lechner-Scott J, Agzarian M (2020) Central vein sign for multiple sclerosis: A systematic review and meta-analysis. Clin Radiol 75(6):479.e9-e15. 10.1016/j.crad.2020.01.011 32143784 10.1016/j.crad.2020.01.011

[34] Chaaban L, Safwan N, Moussa H, El-Sammak S, Khoury SJ, Hannoun S (2022) Central vein sign: A putative diagnostic marker for multiple sclerosis. Acta Neurol Scand 145(3):279–287. 10.1111/ane.13553 34796472 10.1111/ane.13553

[35] Castellaro M, Tamanti A, Pisani AI, Pizzini FB, Crescenzo F, Calabrese M (2020) The use of the central vein sign in the diagnosis of multiple sclerosis: a systematic review and meta-analysis. Diagnostics (Basel). 10.3390/diagnostics10121025 33260401 10.3390/diagnostics10121025 PMC7760678

[36] Hardy TA, Reddel SW, Barnett MH, Palace J, Lucchinetti CF, Weinshenker BG (2016) Atypical inflammatory demyelinating syndromes of the CNS. Lancet Neurol 15(9):967–981. 10.1016/S1474-4422(16)30043-6 27478954 10.1016/S1474-4422(16)30043-6

[37] Tsai KY, Tsai CP, Liao N (2001) Sjögren’s syndrome with central nervous system involvement presenting as multiple sclerosis with failure response to beta-interferon. Eur Neurol 45(1):59–60. 10.1159/000052095 11150847 10.1159/000052095

[38] Keiserman B, da Silva LF, Keiserman MW, von Mühlen CA, Staub HL (2010) Lupoid sclerosis. Rheumatol Int 30(4):431–434. 10.1007/s00296-009-1175-1 19826821 10.1007/s00296-009-1175-1

[39] Borhani-Haghighi A, Kardeh B, Banerjee S, Yadollahikhales G, Safari A, Sahraian MA et al (2019) Neuro-Behcet’s disease: An update on diagnosis, differential diagnoses, and treatment. Mult Scler Relat Disord 39:101906. 10.1016/j.msard.2019.101906 31887565 10.1016/j.msard.2019.101906

[40] Mikaeloff Y, Caridade G, Husson B, Suissa S, Tardieu M, Society NKSGotFN (2007) Acute disseminated encephalomyelitis cohort study: prognostic factors for relapse. Eur J Paediatr Neurol. 11(2):90–95. 10.1016/j.ejpn.2006.11.007 17188007 10.1016/j.ejpn.2006.11.007

[41] Hynson JL, Kornberg AJ, Coleman LT, Shield L, Harvey AS, Kean MJ (2001) Clinical and neuroradiologic features of acute disseminated encephalomyelitis in children. Neurology 56(10):1308–1312. 10.1212/wnl.56.10.1308 11376179 10.1212/wnl.56.10.1308

[42] Tenembaum S, Chitnis T, Ness J, Hahn JS, Group IPMS (2007) Acute disseminated encephalomyelitis. Neurology 68(16 Suppl 2):S23-36. 10.1212/01.wnl.0000259404.51352.7f 17438235 10.1212/01.wnl.0000259404.51352.7f

[43] Pohl D, Alper G, Van Haren K, Kornberg AJ, Lucchinetti CF, Tenembaum S et al (2016) Acute disseminated encephalomyelitis: Updates on an inflammatory CNS syndrome. Neurology 87(9 Suppl 2):S38-45. 10.1212/WNL.0000000000002825 27572859 10.1212/WNL.0000000000002825

[44] Mikaeloff Y, Suissa S, Vallée L, Lubetzki C, Ponsot G, Confavreux C et al (2004) First episode of acute CNS inflammatory demyelination in childhood: prognostic factors for multiple sclerosis and disability. J Pediatr 144(2):246–252. 10.1016/j.jpeds.2003.10.056 14760270 10.1016/j.jpeds.2003.10.056

[45] Li K, Li M, Wen L, Wang Q, Ding X, Wang J (2022) Clinical presentation and outcomes of acute disseminated encephalomyelitis in adults worldwide: systematic review and meta-analysis. Front Immunol 13:870867. 10.3389/fimmu.2022.870867 35757742 10.3389/fimmu.2022.870867 PMC9218070

[46] Schwarz S, Mohr A, Knauth M, Wildemann B, Storch-Hagenlocher B (2001) Acute disseminated encephalomyelitis: a follow-up study of 40 adult patients. Neurology 56(10):1313–1318. 10.1212/wnl.56.10.1313 11376180 10.1212/wnl.56.10.1313

[47] Koelman DL, Mateen FJ (2015) Acute disseminated encephalomyelitis: current controversies in diagnosis and outcome. J Neurol 262(9):2013–2024. 10.1007/s00415-015-7694-7 25761377 10.1007/s00415-015-7694-7

[48] Dagher AP, Smirniotopoulos J (1996) Tumefactive demyelinating lesions. Neuroradiology 38(6):560–565. 10.1007/BF00626098 8880719 10.1007/BF00626098

[49] Lucchinetti CF, Gavrilova RH, Metz I, Parisi JE, Scheithauer BW, Weigand S et al (2008) Clinical and radiographic spectrum of pathologically confirmed tumefactive multiple sclerosis. Brain 131(Pt 7):1759–1775. 10.1093/brain/awn098 18535080 10.1093/brain/awn098 PMC2442427

[50] Altintas A, Petek B, Isik N, Terzi M, Bolukbasi F, Tavsanli M et al (2012) Clinical and radiological characteristics of tumefactive demyelinating lesions: follow-up study. Mult Scler 18(10):1448–1453. 10.1177/1352458512438237 22419670 10.1177/1352458512438237

[51] Siri A, Carra-Dalliere C, Ayrignac X, Pelletier J, Audoin B, Pittion-Vouyovitch S et al (2015) Isolated tumefactive demyelinating lesions: diagnosis and long-term evolution of 16 patients in a multicentric study. J Neurol 262(7):1637–1645. 10.1007/s00415-015-7758-8 25929666 10.1007/s00415-015-7758-8

[52] Nagappa M, Taly AB, Sinha S, Bharath RD, Mahadevan A, Bindu PS et al (2013) Tumefactive demyelination: clinical, imaging and follow-up observations in thirty-nine patients. Acta Neurol Scand 128(1):39–47. 10.1111/ane.12071 23277913 10.1111/ane.12071

[53] Spelman T, Magyari M, Piehl F, Svenningsson A, Rasmussen PV, Kant M et al (2021) Treatment Escalation vs Immediate Initiation of Highly Effective Treatment for Patients With Relapsing-Remitting Multiple Sclerosis: Data From 2 Different National Strategies. JAMA Neurol 78(10):1197–1204. 10.1001/jamaneurol.2021.2738 34398221 10.1001/jamaneurol.2021.2738 PMC8369379

[54] Yeo T, Probert F, Jurynczyk M, Sealey M, Cavey A, Claridge TDW et al (2019) Classifying the antibody-negative NMO syndromes: Clinical, imaging, and metabolomic modeling. Neurol Neuroimmunol Neuroinflamm 6(6):e626. 10.1212/NXI.0000000000000626 31659123 10.1212/NXI.0000000000000626 PMC6865851

[55] Juryńczyk M, Klimiec-Moskal E, Kong Y, Hurley S, Messina S, Yeo T, Jenkinson M, Leite MI, Palace J (2022) Elucidating distinct clinico-radiologic signatures in the borderlasnd between neuromyelitis optica and multiple sclerosis. J Neurol 269(1):269–279. 10.1007/s00415-021-10619-1 34043042 10.1007/s00415-021-10619-1 PMC8738499

