# Supplementary material for: Beyond dissemination criteria: lesion-based longitudinal MRI of atypical demyelinating lesions at the boundary of multiple sclerosis
Source: J Neurol. 2026 Jul 1;273(7):438. doi: 10.1007/s00415-026-13951-6 (PMC13323187; doi:10.1007/s00415-026-13951-6)
Supplement: Supplementary file 1 — Supplementary file1 (DOCX 25 KB) [file 415_2026_13951_MOESM1_ESM.docx]

***Supplementary Table***

| **Supplementary Table 1. Clinical and demographic characteristics of 40 patients with radiologically typical MS patients** | | | | | | | | | | | | |  |  |  |  |  |  |  |  |
| --- | --- | --- | --- | --- | --- | --- | --- | --- | --- | --- | --- | --- | --- | --- | --- | --- | --- | --- | --- | --- |
| **No** | **Age group** | **sex** | **OCB** | **MBP** | **IgG index** | **Spinal cord lesions** | **ON** | **Gd** | **EDSS** | **treatment** | **lesion volume (mm3)** |  |  |  |  |  |  |  |  |  |
| 1 | 40 | F | N/A | N/A | N/A | - | + | N/A | 7 | NTZ | 746.543065 |  |  |  |  |  |  |  |  |  |
| 2 | 40 | F | - | - | 0.5≦ | C, T | - | - | 5.5 | NTZ | N/A |  |  |  |  |  |  |  |  |  |
| 3 | 40 | F | - | - | 0.5≦ | - | + | - | 2 | OFM | 409.761986 |  |  |  |  |  |  |  |  |  |
| 4 | 40 | F | - | N/A | 0.5≦ | C, T | + | - | 6 | DMF | N/A |  |  |  |  |  |  |  |  |  |
| 5 | 50 | F | + | N/A | 0.5≦ | - | + | N/A | 3.5 | NTZ | 1329.21937 |  |  |  |  |  |  |  |  |  |
| 6 | 30 | F | - | - | 0.5≦ | C | - | - | 0 | NTZ | 99.6589658 |  |  |  |  |  |  |  |  |  |
| 7 | 50 | M | + | + | 0.5≦ | T | - | - | 6 | OFM | N/A |  |  |  |  |  |  |  |  |  |
| 8 | 40 | F | - | - | 0.5≦ | C | - | - | 1 | NTZ | 131.847067 |  |  |  |  |  |  |  |  |  |
| 9 | 50 | M | + | - | 0.5≦ | C | - | - | 4 | NTZ | 957.460424 |  |  |  |  |  |  |  |  |  |
| 10 | 50 | F | - | N/A | 0.5> | C | - | - | 6.5 | NTZ | 598.770851 |  |  |  |  |  |  |  |  |  |
| 11 | 20 | M | - | N/A | 0.5≦ | C, T | + | - | 1.5 | NTZ | 147.438049 |  |  |  |  |  |  |  |  |  |
| 12 | 40 | M | - | N/A | 0.5≦ | - | - | - | 5.5 | NTZ | N/A |  |  |  |  |  |  |  |  |  |
| 13 | 30 | F | N/A | N/A | N/A | C, T | + | - | 4 | NTZ | 862.439473 |  |  |  |  |  |  |  |  |  |
| 14 | 50 | F | - | N/A | 0.5≦ | C | + | - | 3.5 | NTZ | 111750.5246 |  |  |  |  |  |  |  |  |  |
| 15 | 40 | F | + | N/A | 0.5≦ | - | + | - | 2 | NTZ | 277.966929 |  |  |  |  |  |  |  |  |  |
| 16 | 50 | F | N/A | N/A | N/A | C | - | - | 3.5 | NTZ | 2880.68327 |  |  |  |  |  |  |  |  |  |
| 17 | 40 | F | + | N/A | 0.5≦ | C | - | + | 4.5 | NTZ | 176.22257 |  |  |  |  |  |  |  |  |  |
| 18 | 50 | F | - | N/A | 0.5≦ | C | - | - | 6 | OFM | N/A |  |  |  |  |  |  |  |  |  |
| 19 | 30 | F | + | N/A | N/A | C | - | + | 1 | NTZ | 308.523185 |  |  |  |  |  |  |  |  |  |
| 20 | 40 | F | + | - | 0.5≦ | T | - | N/A | 3.5 | NTZ | N/A |  |  |  |  |  |  |  |  |  |
| 21 | 50 | F | - | N/A | N/A | C, T | - | + | 7 | NTZ | 433.206558 |  |  |  |  |  |  |  |  |  |
| 22 | 50 | F | - | N/A | 0.5≦ | - | + | - | 1.5 | NTZ | N/A |  |  |  |  |  |  |  |  |  |
| 23 | 50 | F | - | - | 0.5> | - | - | + | 2 | NTZ | N/A |  |  |  |  |  |  |  |  |  |
| 24 | 50 | M | - | N/A | 0.5> | C | + | - | 4.5 | NTZ | N/A |  |  |  |  |  |  |  |  |  |
| 25 | 30 | M | N/A | N/A | N/A | - | - | - | 1.5 | NTZ | 77.971 |  |  |  |  |  |  |  |  |  |
| 26 | 50 | F | N/A | N/A | N/A | - | + | - | 1 | NTZ | N/A |  |  |  |  |  |  |  |  |  |
| 27 | 40 | F | N/A | N/A | 0.5≦ | T | - | N/A | 2 | fingolimod | 221.121316 |  |  |  |  |  |  |  |  |  |
| 28 | 40 | F | - | N/A | N/A | - | - | - | 1.5 | NTZ | 65.2045023 |  |  |  |  |  |  |  |  |  |
| 29 | 20 | M | + | - | 0.5≦ | C, T | - | - | 2 | DMF | 347.137451 |  |  |  |  |  |  |  |  |  |
| 30 | 20 | M | + | - | 0.5≦ | T | - | N/A | 6 | NTZ+PSL | 811.222385 |  |  |  |  |  |  |  |  |  |
| 31 | 40 | F | - | N/A | N/A | - | - | - | 2 | DMF | N/A |  |  |  |  |  |  |  |  |  |
| 32 | 30 | F | + | N/A | 0.5≦ | C, T | + | + | 2 | NTZ | 179.165676 |  |  |  |  |  |  |  |  |  |
| 33 | 30 | F | + | N/A | 0.5≦ | - | - | - | 4.5 | DMF | 69.48443 |  |  |  |  |  |  |  |  |  |
| 34 | 20 | F | + | - | 0.5≦ | - | + | - | 4 | NTZ | N/A |  |  |  |  |  |  |  |  |  |
| 35 | 50 | F | + | + | 0.5≦ | - | - | + | 0 | OFM | N/A |  |  |  |  |  |  |  |  |  |
| 36 | 40 | F | - | N/A | N/A | C, T | + | - | 4.5 | none | N/A |  |  |  |  |  |  |  |  |  |
| 37 | 40 | F | + | N/A | 0.5≦ | - | - | - | 1 | DMF | N/A |  |  |  |  |  |  |  |  |  |
| 38 | 40 | F | + | N/A | 0.5≦ | C | - | - | 2 | NTZ | 142.335892 |  |  |  |  |  |  |  |  |  |
| 39 | 40 | M | - | N/A | 0.5≦ | C | - | N/A | 3.5 | NTZ | 2198.202389 |  |  |  |  |  |  |  |  |  |
| 40 | 30 | F | + | - | 0.5≦ | C | - | N/A | 1 | PSL | 581.985011 |  |  |  |  |  |  |  |  |  |
